# Sex Differences in Odds of Brain Metastasis and Outcomes by Brain Metastasis Status after Advanced Melanoma Diagnosis

**DOI:** 10.3390/cancers16091771

**Published:** 2024-05-03

**Authors:** Gino Cioffi, Mustafa S. Ascha, Kristin A. Waite, Mantas Dmukauskas, Xiaoliang Wang, Trevor J. Royce, Gregory S. Calip, Timothy Waxweiler, Chad G. Rusthoven, Brian D. Kavanagh, Jill S. Barnholtz-Sloan

**Affiliations:** 1 Trans Divisional Research Program, Division of Cancer Epidemiology and Genetics, National Cancer Institute, Bethesda, MD 20892,USAmantas.dmukauskas@nih.gov (M.D.); 2 Flatiron Health, Inc., New York, NY 10013, USAtrevor.royce@gmail.com (T.J.R.);; 3 Department of Radiation Oncology, Wake Forest University School of Medicine, Winston-Salem, NC 27157, USA; 4 Titus Family Department of Clinical Pharmacy, University of Southern California, Los Angeles, CA 90089, USA; 5 Department of Radiation Oncology, University of Colorado Cancer Center, Anschutz Medical Campus, Aurora, CO 80045, USA; 6 Center for Biomedical Informatics & Information Technology, National Cancer Institute, Bethesda, MD 20892,USA

**Keywords:** brain metastasis, melanoma, outcomes, sex differences

## Abstract

**Simple Summary:**

Sex differences in cancer are well-established. However, less is known about sex differences in the diagnosis of brain metastasis and outcomes among patients with advanced melanoma. Using the nationwide Flatiron Health electronic health record-derived de-identified database, this study showed that males had greater odds of brain metastasis, and poorer real-world overall survival compared to females among those with brain metastasis, while there were no sex differences in clinical outcomes for those with advanced melanoma without brain metastasis.

**Abstract:**

Sex differences in cancer are well-established. However, less is known about sex differences in diagnosis of brain metastasis and outcomes among patients with advanced melanoma. Using a United States nationwide electronic health record-derived de-identified database, we evaluated patients diagnosed with advanced melanoma from 1 January 2011–30 July 2022 who received an oncologist-defined rule-based first line of therapy (*n* = 7969, 33% female according to EHR, 35% w/documentation of brain metastases). The odds of documented brain metastasis diagnosis were calculated using multivariable logistic regression adjusted for age, practice type, diagnosis period (pre/post-2017), ECOG performance status, anatomic site of melanoma, group stage, documentation of non-brain metastases prior to first-line of treatment, and BRAF positive status. Real-world overall survival (rwOS) and progression-free survival (rwPFS) starting from first-line initiation were assessed by sex, accounting for brain metastasis diagnosis as a time-varying covariate using the Cox proportional hazards model, with the same adjustments as the logistic model, excluding group stage, while also adjusting for race, socioeconomic status, and insurance status. Adjusted analysis revealed males with advanced melanoma were 22% more likely to receive a brain metastasis diagnosis compared to females (adjusted odds ratio [aOR]: 1.22, 95% confidence interval [CI]: 1.09, 1.36). Males with brain metastases had worse rwOS (aHR: 1.15, 95% CI: 1.04, 1.28) but not worse rwPFS (adjusted hazard ratio [aHR]: 1.04, 95% CI: 0.95, 1.14) following first-line treatment initiation. Among patients with advanced melanoma who were not diagnosed with brain metastases, survival was not different by sex (rwOS aHR: 1.06 [95% CI: 0.97, 1.16], rwPFS aHR: 1.02 [95% CI: 0.94, 1.1]). This study showed that males had greater odds of brain metastasis and, among those with brain metastasis, poorer rwOS compared to females, while there were no sex differences in clinical outcomes for those with advanced melanoma without brain metastasis.

## 1. Introduction

Despite aggressive multi-modal therapies, the survival rates for cancer patients with brain metastases (BrM) remains generally poor. Over the last decade, tremendous progress has been made in uncovering the molecular genetics of malignant primary brain tumors, allowing for identification of key hallmark alterations associated with accuracy of diagnosis and prognosis. This same progress has not been made in BrM. BrM continues to be one of the major complications of advanced melanoma and is the most common cause of melanoma deaths [1,2]. The median overall survival of a patient with melanoma that has developed BrM is less than 2 years.

Sex is an important factor which has been shown to influence disease development, progression, and clinical outcomes. Sexual differentiation results in sex differences in cellular and systems biology [3,4,5]. These sex-specific differences produce sexually dimorphic traits, including metabolism and disease risk [6,7]. Sex disparities in cancer incidence, survival, and prevalence have been well established for a variety of cancers, including brain tumors. Not only do males develop cancers 20% more often than females, males also have poorer responses to therapy, as measured by poorer progression-free and overall survival determinations compared to females [8,9]. Our group has shown that the incidence of glioblastoma (GBM) is higher in males through all stages of life [10]. In addition, females with GBM have a statistically significant overall survival advantage after adjustment for age, Karnofsky performance status, extent of resection, and receipt of standard of care [11]. Additionally, sex differences in melanoma have been well-documented, in which similarly, males have higher incidence and worse survival outcomes compared to females [8,12,13,14]. The exact biological mechanisms underlying these sex differences remain to be determined.

While sex differences in primary tumors, such as GBM and melanoma, have been well established, less is known about sex differences in the diagnosis of BrM and clinical outcomes among patients with advanced melanoma. This study aims to evaluate potential sex differences in the odds of brain metastasis and survival after brain metastasis among patients with advanced melanoma.

## 2. Methods

Study Design: This study was a retrospective observational cohort study using the nationwide Flatiron Health electronic health record (EHR)-derived de-identified database. Institutional Review Board approval of the study protocol was obtained prior to the conducting of the study and included a waiver of informed consent. We followed the Strengthening the Reporting of Observational Studies in Epidemiology (STROBE) reporting guideline. The Flatiron Health database is a longitudinal database, comprising de-identified patient-level structured and unstructured data, curated via technology-enabled abstraction [15,16].

Patient Selection: The study period was 1 January 2011 through 31 December 2022. During this time period, the de-identified patient data originated from approximately 280 cancer clinics (~800 sites of care) in the United States (US). Care sites consisted of community oncology and academic medical center clinics. The study population included patients diagnosed with advanced melanoma from 1 January 2011 through 1 July 2022 who had at least two documented EHR visits on or after 1 January 2011, documented treatment with a first-line therapy, and a notation of their sex associated with the record. Individuals were selected based on ICD codes (ICD-9: 172.x, ICD-10: C43x, or D03x) and a determination of pathologic stages III or IV at initial diagnosis or at locoregional or distant recurrence, and diagnosis of advanced melanoma was confirmed via abstraction. Diagnosis date was limited to six months prior to data cutoff (31 December 2022), in order to allow for a potential minimum of six months’ patient observation.

Study Variables and Endpoints: The primary predictors of interest in this study were sex and BrM status. Sex was typically documented by clinicians upon patient intake and is a structured data element available in the data; it is assumed to be the sex assigned at birth. BrM status was determined through abstraction based on clinical and pathologic statements that confirm the metastatic site and date of advanced melanoma diagnosis. The date of BrM diagnosis was determined as the earliest date that a patient showed evidence of BrM from a radiology scan, pathology report, or physician statement. Date of BrM diagnosis was recorded at the month-level; thus, estimates of times to events using BrM diagnosis were also at the month-level. 

Patient-level clinical and demographic characteristics were identified using both structured and unstructured data. Technology-enabled abstraction was used to extract relevant data from unstructured data. Clinical and demographic data included age at advanced melanoma diagnosis, SES quintile at the Census block group level, anatomic site of melanoma as determined by structured diagnosis codes, group stage at initial melanoma diagnosis, diagnosis of non-brain metastases prior to the first line of systemic treatment, and BRAF mutation status (positive/negative; 30 days prior, or up to 60 days after advanced diagnosis date). Additionally, Eastern Cooperative Oncology Group (ECOG) performance status (PS) was determined from the structured data and extracted using natural language processing, as determined at around the time of first-line treatment (30 days prior to up to 7 days after first-line initiation). Age at advanced melanoma diagnosis was subject to de-identification requirements such that the earliest recorded birth year was 85 years prior to data cutoff year of 1937. 

Endpoints included real-world overall survival (rwOS) and real-world progression-free survival (rwPFS) [17]. Mortality was identified using structured and unstructured EHR documentation supplemented with external commercial and U.S. Social Security Death Index data. This composite mortality endpoint has been validated with high agreement and accuracy against the National Death Index [18]. 

Analyses: Descriptive statistics of baseline characteristics were summarized across the overall population, as well as stratified by sex and BrM diagnosis. Differences in distributions were assessed with an χ^2^ test. Logistic regression was used to estimate the adjusted and unadjusted odds of BrM diagnosis by sex in the overall population.

Cox proportional hazards regression was used to estimate the adjusted and unadjusted hazard of mortality, as well as the hazard of disease progression or mortality, as indexed to first-line of therapy start date among all patients. The rwOS was defined as ending on date of death, censoring at last confirmed activity. The rwPFS was defined as ending on the first clinician documentation of disease progression or date of death, censoring at the date of the last clinic note [19].

For each adjusted model, adjusted covariates were identified using forward stepwise variable selection, measured by change in Akaike information criterion (AIC), for which candidate covariates included the following: age at diagnosis (categorical: 0–34, 35–49, 50–64, 65–74, and 75+), race/ethnicity (Asian, Black or African American, Hispanic or Latino, White, Other, or Unknown), practice type (academic or community), year of advanced melanoma diagnosis (before 2017, during or after 2017), insurance coverage at advanced diagnosis (a year prior to, or up to 30 days after advanced diagnosis date), SES index (quintile), ECOG PS (0, 1, 2+, or unknown), anatomic site of melanoma per ICD codes, group stage at initial diagnosis, record of metastases other than brain prior to the start of the first line of treatment, and BRAF biomarker status. Year of advanced melanoma diagnosis was included as a categorical variable to account for potential era effects associated with the use of checkpoint inhibitor therapy for advanced melanoma. For the logistic regression analysis, candidate covariates that were dropped from the final model were race, insurance at advanced diagnosis, and SES index. For the Cox proportional hazards models assessing rwOS, the candidate covariate dropped from the model was group stage at initial diagnosis, for both those with and those without documented BrM. For the Cox proportional hazard models assessing rwPFS, candidate covariates that were dropped from the final model for those with documented BrM included race, practice type, SES index, anatomic site of melanoma (ever), group stage at initial diagnosis, and history of metastases other than brain prior to 1L start, while covariates dropped from the final model for those without documented BrM included practice type, as well as history of BRAF status. Cox regression models estimating rwOS and rwPFS for patients with documented BrM additionally included a BrM diagnosis as a time-varying covariate. 

A reverse Kaplan–Meier analysis was performed, in which loss to follow-up was recorded as the ‘event’ and death as ‘censoring’ to evaluate differences in follow-up time between those who did and did not develop BrM.

## 3. Results

Overall, there were 7969 patients with advanced melanoma identified, with 2794 (35%) having documentation of BrM, and there were a higher percentage of male patients with BrM, compared to those without BrM (69% vs. 66% respectively, *p* = 0.003) (Supplemental Table S1). Descriptive statistics stratified by sex, presented separately for patients with BrM and without documented BrM, are described in Table 1. There was a notable difference in age distribution by sex, with a higher proportion of older individuals among the male patients with BrM (65–74 yrs: 31% male vs. 24% female, 75+ yrs: 21% male vs. 15% female, *p* < 0.001) and among male patients without BrM (65–74: 29% male vs. 27% female, 75+: 35% male vs. 29% female, *p* < 0.001). Additionally, sex differences in Medicare coverage were also observed, in that males were more likely to have Medicare coverage than were females, both among patients with BrM (18% male vs. 15% female, *p* = 0.036) and among those with no documented BrM (22% male vs. 20% female, *p* = 0.034). There was a significant difference in the distribution of SES index by sex among non-BrM patients, with a higher percentage of lowest-SES individuals among females (12%) compared to males (9.3%, *p* = 0.009). Additionally, there was a sex difference in the distribution of patients with metastases outside of the brain prior to the start of first line of treatment among non-BrM patients (74% male vs. 71% female, *p* = 0.012). Across both groups, the *BRAF* mutation was more common in males than females (BrM: 39% male vs. 36% female, *p* = 0.003, non-BrM: 29% male vs. 25% female, *p* < 0.001).

**Figure 1:**
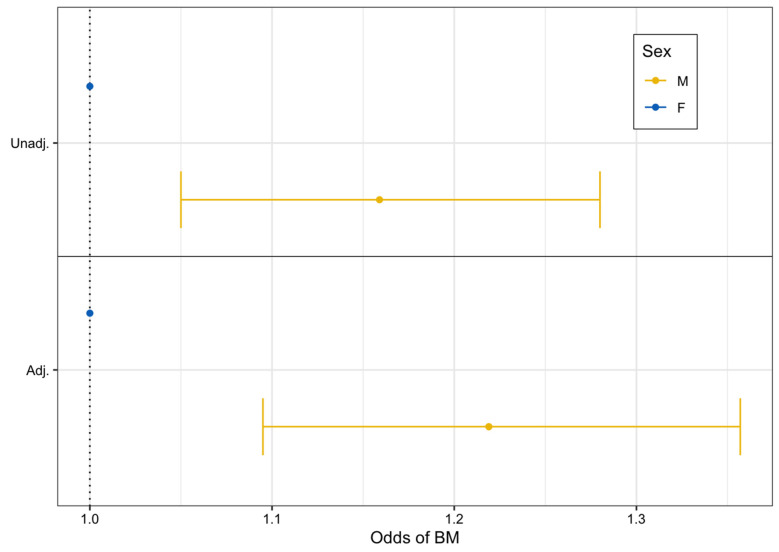
Unadjusted and adjusted logistic regression forest plot assessing the odds of brain metastasis after advanced melanoma diagnosis by sex (2011–2022).

From unadjusted logistic regression modeling, we found that males had 16% higher odds of developing BrM compared to females (unadjusted OR: 1.16, 95% CI: 1.05–1.28, *p* = 0.003). After adjustment for additional factors, males still had statistically significantly higher odds of BrM development compared to females (adjusted OR: 1.22, 95% CI: 1.10–1.36, *p* < 0.001) (Figure 1).

**Figure 2:**
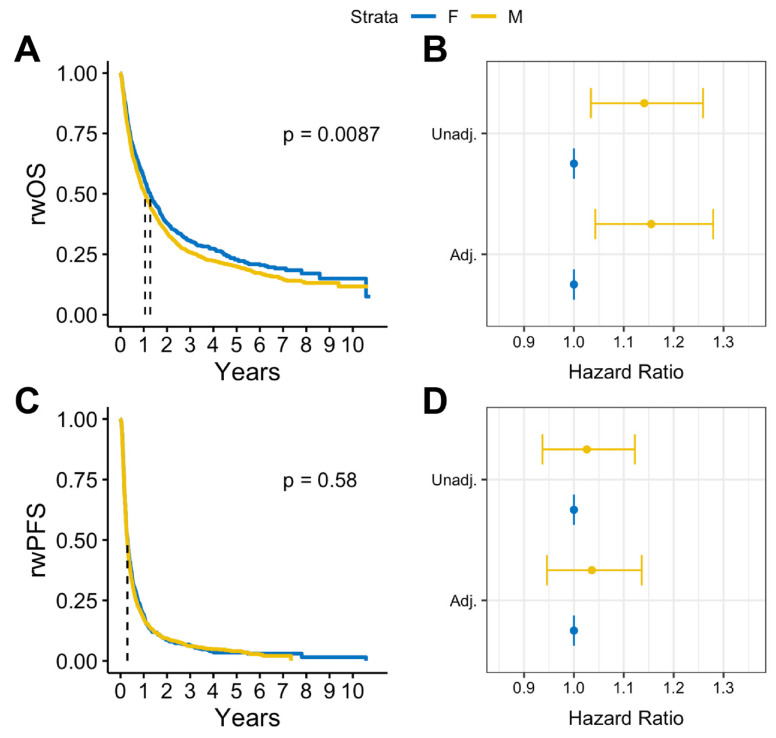
(**A**) Kaplan–Meier survival curves and (**B**) Cox proportional hazards forest plots for real-world overall survival and (**C**) Kaplan-Meier survival curves and (**D**) Cox proportional hazard forest plots for progression-free survival for advanced melanoma cases with brain metastases (2011–2022).

**Figure 3:**
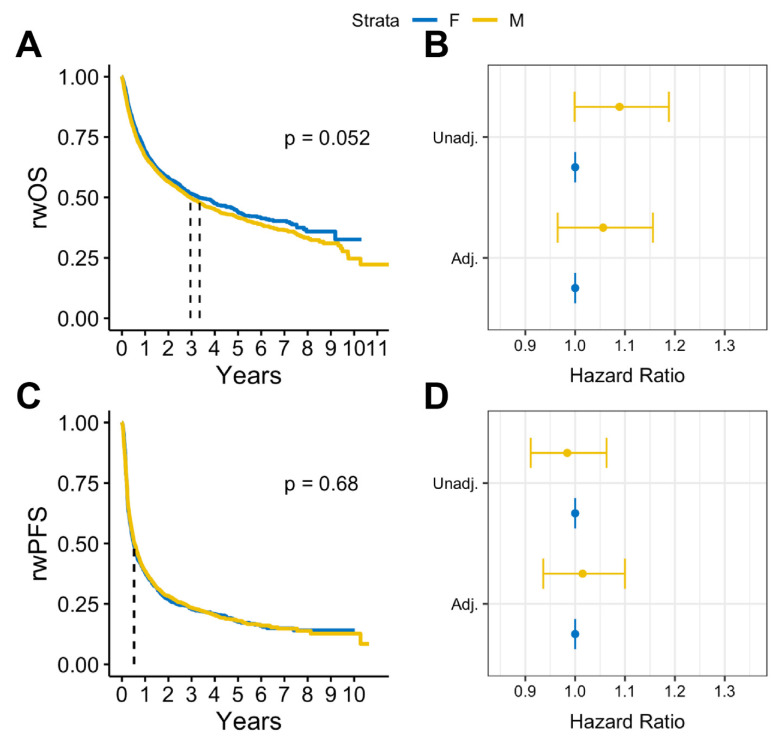
(**A**) Kaplan Meier survival curves and (**B**) Cox proportional hazards forest plots for real-world overall survival and (**C**) Kaplan-Meier survival curves and (**D**) Cox proportional hazards forest plots for progression-free survival for advanced melanoma cases without brain metastases (2011–2022).

In survival analysis, we found that among patients with BrM, males had statistically significantly lower median rwOS than females (13 months vs. 15 months, log rank *p* = 0.009), but there was no observed difference in rwPFS (log rank *p* = 0.58) (Figure 2A,B). Similar results were observed after adjusting for covariates. Among patients with BrM, males had worse rwOS compared to females (adjusted HR: 1.18, 95% CI:1.05-1.32, *p* = 0.005) when adjusted for select covariates (Figure 2C). No statistically significant differences by sex were observed on rwPFS (Figure 2D). Overall, in patients with advanced melanoma without documented BrM, there were no statistically significant sex differences in either rwOS or rwPFS observed (Figure 3).

From the reverse Kaplan–Meier analysis, it was found that individuals who developed BrM were 24% less likely to be lost to follow-up at any given time compared to those who did not develop BrM (HR: 0.76, 95% CI: 0.71–0.82, *p* < 0.001). 

## 4. Discussion

In this study, we report that among patients with advanced melanoma, males are more likely to develop BrM, as compared to females. Further, among patients diagnosed with BrM, males have worse rwOS outcomes compared to females. In contrast, there were no observed differences in rwPFS. Our results suggest that among patients with advanced melanoma, there may be sex disparities in the development of BrM and survival after BrM development.

The prevalent use of tanning beds, and subsequent UV exposure, most commonly observed in young, adolescent females, has been implicated in increased melanoma incidence in younger women [20]. Indeed, indoor tanning has been linked as a contributing factor for the increased melanoma rates in younger women when compared to men [21]. Given the increased risk of metastases at a younger primary melanoma diagnosis, and higher female incidence of melanoma in this earlier age demographic, one may expect BrM risk to be greater in females. However, based on our findings, the odds of BrM development are significantly greater for males than females, when adjusted for risk factors like age at advanced melanoma diagnosis, which may indicate potential variations in behaviors or underlying biological mechanisms that could be driving this difference. Males tend to utilize primary care less than females do, allowing tumors time to grow and deepen [22,23]. Further, skin awareness, a known activity associated with the discovery of thinner melanoma lesions and a significant decrease in melanoma mortality [24], is more prevalent in females than males [25,26,27]. These health behaviors in males have thus been suggested to contribute to the prevalence of later-tumor-stage melanoma tumors in males.

Sex differences were also observed in overall survival, with males having significantly worse rwOS than females among patients with advanced melanoma who developed BrM. Survival differences by sex have been well documented in melanoma [8,12,13]. However, the majority of these studies do not focus on advanced melanoma or analysis relevant to the metastasis to the brain. A study by Enniga et al. found that sex differences in melanoma survival have variations by stage, with significant differences occurring more often in earlier stages (I–II) [14]. A small proportion of the patients in our study were classified as stage II or lower at initial diagnosis (26%), and at the time of the study had recurrent disease with a pathological stage of III or IV, which may explain the lack of significant sex difference in survival among patients who did not develop a BM. The possibility that males are diagnosed later in the course of disease could contribute to a “lead time bias” that suggests males have shorter survival, whether overall or progression-free, despite potentially there being no such difference when indexing to comparable points in the natural history of disease. Additionally, the observed median survival differences by sex among those with BrM, while statistically significant, are small (2 months). However, given the aggressive prognosis of metastatic melanoma, even incremental changes in overall survival are clinically relevant. 

Several factors analyzed in this study have been previously reported to impact the development of BrM, including *BRAF* mutation status. The *BRAF* mutation has been shown to be associated with distant metastases in advanced melanoma and other cancers [28,29,30]. Earlier works by others have demonstrated that advanced melanoma patients with *BRAF* mutations not only have an increased risk of developing BrM, but also have a shorter disease-free interval from primary diagnosis to diagnosis of BrM [31]. Similar results were found here with regard to advanced melanoma patients who progressed to BrM, as patients with *BRAF* mutation had a higher proportion of BrM development than those without a mutation. While advanced melanoma patients with *BRAF* mutations have increased risk for BrM, those patients with BrM who are *BRAF* mutation-positive have a better survival than BrM patients who are mutation-negative. This is likely due to the availability of targeted therapies [32]. The most common BRAF mutation is V600E [28]; however, the V600K mutation has been associated with males and those patients of older age [33]. While we did observe a sex bias in the distribution of *BRAF* status, with males having a higher percentage of mutation-positive cases, when adjusted for *BRAF* status sex was still found to be a significant predictor of BrM development. Further, advanced melanoma patients with the BRAF V6000K mutation have poorer survival when compared to those with the V600E mutation [33]. Thus, it may be plausible to postulate given this dataset that perhaps the observation of poorer survival in males is related to a higher percentage of patients having the V600K mutation. Due to limitations within the data, we are unable to adjust for BRAF mutation type.

Sex-specific signatures in the tumor microenvironment have been reported globally [34] as well as within the brain [35,36,37,38]. In primary brain tumors, research has shown that sex differences in the tumor microenvironment, particularly in immune cell composition, contribute to sex differences in survival [39]. Additionally, transcriptomic analysis has demonstrated differences in transcription differences between males and females that correspond to survival [39,40]. While these studies have all been performed in primary brain tumors, it remains a possibility that similar sex differences in the metastatic brain microenvironment contribute to the significant survival differences observed among patients who developed BrM. This remains to be investigated and is beyond the scope of this analysis.

To our knowledge, this study is one of the largest contemporary studies assessing the association between sex and BrM, in addition to subsequent survival outcomes among real-world patients with advanced melanoma in the US. We leveraged a large nationwide cohort of patients with curated and harmonized data which included abstraction of confirmed advanced melanoma diagnosis, demographic and clinical risk factors, and progression. This allowed us to not only evaluate diagnoses of BrM, but also both rwPFS and rwOS outcomes. Long-term longitudinal follow-up and assessment data throughout the patient treatment journey made this study feasible.

The findings of our work must be considered within the context of the study’s limitations. First, sex is documented as structured data in the EHR data, and likely reflects the patient’s sex at birth. Second, although we were able to adjust for several demographic and clinical factors, as is common with observational studies utilizing real-world data, certain relevant characteristics were not available, such as UV exposure level. The results from this study may be impacted by unmeasured clinical, demographic, or other confounding factors. Additionally, individuals with documented BrM were followed up longer than those without BrM documentation, which suggests that patients without BrM might have been more likely to be lost to follow-up, resulting in misclassification of BrM among those patients. Lastly, this study is U.S.-based, and our results may not be generalizable to patients who received care outside the U.S.

This study aimed to investigate potential sex differences in BrM development, and survival after BrM development, among patients with advanced melanoma. It was observed that males have higher odds of BrM development, and a higher risk of death after BrM development, compared to females, as adjusted for known risk factors such as age and BRAF mutation status. Studies such as these provide crucial insights on differences in disease burden, which can help drive efforts to improve patient outcomes.

## Figures and Tables

**Table 1:** Distribution of demographic and clinical characteristics for patients with advanced melanoma according to documentation of brain metastases and sex (2011–2022).

Characteristic	No Documentation of BrM			Has Documentation of BrM		
	Female, *n* = 1752 (34%)	Male, *n* = 3423 (66%)	*p*-Value	Female, *n* = 856 (31%)	Male, *n* = 1938 (69%)	*p*-Value
**Age at Diagnosis**			<0.001			<0.001
0–34	70 (4.0%)	74 (2.2%)		47 (5.5%)	74 (3.8%)	
35–49	207 (12%)	252 (7.4%)		169 (20%)	239 (12%)	
50–64	509 (29%)	892 (26%)		305 (36%)	626 (32%)	
65–74	465 (27%)	1006 (29%)		203 (24%)	592 (31%)	
75+	501 (29%)	1199 (35%)		132 (15%)	407 (21%)	
**Race**			0.013			0.4
Asian	5 (0.3%)	8 (0.2%)		6 (0.7%)	4 (0.2%)	
Black or African American	17 (1.0%)	10 (0.3%)		5 (0.6%)	9 (0.5%)	
Hispanic or Latino	2 (0.1%)	0 (0%)		0 (0%)	1 (<0.1%)	
White	1459 (83%)	2899 (85%)		716 (84%)	1618 (83%)	
Other Race	124 (7.1%)	230 (6.7%)		58 (6.8%)	139 (7.2%)	
Unknown	145 (8.3%)	276 (8.1%)		71 (8.3%)	167 (8.6%)	
**Practice Type**			0.04			0.5
Academic	470 (27%)	899 (26%)		253 (30%)	541 (28%)	
Community	1247 (71%)	2485 (73%)		592 (69%)	1364 (70%)	
Both	35 (2.0%)	39 (1.1%)		11 (1.3%)	33 (1.7%)	
**Year of Advanced Diagnosis**			>0.9			0.08
Before 2017	765 (44%)	1493 (44%)		464 (54%)	981 (51%)	
During or After 2017	987 (56%)	1930 (56%)		392 (46%)	957 (49%)	
**Insurance at Advanced Diagnosis**			0.034			0.036
Commercial Health Plan	291 (17%)	515 (15%)		200 (23%)	392 (20%)	
Medicaid	29 (1.7%)	41 (1.2%)		32 (3.7%)	35 (1.8%)	
Medicare (Any Program)	352 (20%)	766 (22%)		132 (15%)	343 (18%)	
Other Government-Sponsored/Patient Assistance/Self-Pay	25 (1.4%)	87 (2.5%)		15 (1.8%)	35 (1.8%)	
Multiple Documented	670 (38%)	1244 (36%)		276 (32%)	644 (33%)	
Multiple + Other Payer—Type Unknown	224 (13%)	431 (13%)		100 (12%)	248 (13%)	
Other Payer—Type Unknown	40 (2.3%)	76 (2.2%)		23 (2.7%)	61 (3.1%)	
Unknown	121 (6.9%)	263 (7.7%)		78 (9.1%)	180 (9.3%)	
**Block Group SES index (2015–2019)**			0.009			0.14
1—Lowest SES	207 (12%)	318 (9.3%)		107 (12%)	183 (9.4%)	
2	292 (17%)	502 (15%)		144 (17%)	339 (17%)	
3	362 (21%)	700 (20%)		162 (19%)	369 (19%)	
4	363 (21%)	793 (23%)		169 (20%)	431 (22%)	
5—Highest SES	371 (21%)	782 (23%)		188 (22%)	400 (21%)	
Unknown	157 (9.0%)	328 (9.6%)		86 (10%)	216 (11%)	
**ECOG Performance Score**			0.6			0.081
0	712 (41%)	1447 (42%)		283 (33%)	696 (36%)	
1	485 (28%)	927 (27%)		270 (32%)	609 (31%)	
2+	200 (11%)	358 (10%)		127 (15%)	223 (12%)	
Unknown	355 (20%)	691 (20%)		176 (21%)	410 (21%)	
**Anatomic Site of Melanoma**			NA			NA
Head and Neck	224 (13%)	862 (25%)		109 (13%)	375 (19%)	
Lower Limb	235 (13%)	422 (12%)		109 (13%)	195 (10%)	
Upper Limb	338 (19%)	237 (6.9%)		92 (11%)	69 (3.6%)	
Overlapping	63 (3.6%)	128 (3.7%)		47 (5.5%)	114 (5.9%)	
Truncal	368 (21%)	754 (22%)		181 (21%)	471 (24%)	
Overlapping/Head and Neck	14 (0.8%)	41 (1.2%)		10 (1.2%)	25 (1.3%)	
Truncal/Head and Neck	17 (1.0%)	63 (1.8%)		9 (1.1%)	28 (1.4%)	
Truncal/Lower Limb	26 (1.5%)	61 (1.8%)		14 (1.6%)	28 (1.4%)	
Other	136 (7.8%)	209 (6.1%)		70 (8.2%)	138 (7.1%)	
Unspecified	324 (18%)	639 (19%)		212 (25%)	488 (25%)	
Unknown	7 (0.4%)	7 (0.2%)		3 (0.4%)	7 (0.4%)	
**Group Stage at Advanced Diagnosis**			0.008			NA
0	4 (0.2%)	15 (0.4%)		3 (0.4%)	11 (0.6%)	
I	164 (9.4%)	264 (7.7%)		83 (9.7%)	170 (8.8%)	
II	282 (16%)	607 (18%)		122 (14%)	306 (16%)	
III	550 (31%)	986 (29%)		178 (21%)	360 (19%)	
IV	439 (25%)	977 (29%)		273 (32%)	690 (36%)	
Not Documented	313 (18%)	574 (17%)		197 (23%)	401 (21%)	
**Had Metastases Outside of the Brain prior to 1L Start**			0.012			0.7
Yes	1247 (71%)	2548 (74%)		783 (91%)	1780 (92%)	
No	505 (29%)	875 (26%)		73 (9%)	158 (8%)	
**History of Positive BRAF Status**			<0.001			0.003
Yes	503 (29%)	841 (25%)		336 (39%)	701 (36%)	
No	587 (34%)	1304 (38%)		238 (28%)	664 (34%)	
Unknown	662 (38%)	1278 (37%)		282 (33%)	573 (30%)	

## Data Availability

The data that support the findings of this study were originated by Flatiron Health, Inc. Requests for data sharing by license or by permission for the specific purpose of replicating results in this manuscript can be submitted to PublicationsDataAccess@flatiron.com.

## Supplementary Materials

The following supporting information can be downloaded at https://www.mdpi.com/article/10.3390/cancers16091771/s1. Supplemental Table S1: Distribution of demographic and clinical characteristics for patients with advanced melanoma by documentation of brain metastases (2011–2022).
