# Supplementary material for: Sex Differences in Odds of Brain Metastasis and Outcomes by Brain Metastasis Status after Advanced Melanoma Diagnosis
Source: Cancers (Basel). 2024 May 3;16(9):1771. doi: 10.3390/cancers16091771 (PMC11083203; doi:10.3390/cancers16091771)
Supplement: Supplementary file 1 [file cancers-16-01771-s001.zip › cancers-2981546-supplementary.pdf]

Supplemental Table 1: Distribution of demographic and clinical characteristics for patients with advanced melanoma by documentation of brain metastases (2011-2022).

Characteristic	Overall	Brain Metastasis Status		
	N = 7,969	No Documentation of BrM, n = 5,175 (65%)	Has Documentation of BrM, n = 2,794 (35%)	p-value
**Age at Diagnosis**				<0.001
0-34	265 (3.3%)	144 (2.8%)	121 (4.3%)	
35-49	867 (11%)	459 (8.9%)	408 (15%)	
50-64	2,332 (29%)	1,401 (27%)	931 (33%)	
65-74	2,266 (28%)	1,471 (28%)	795 (28%)	
75+	2,239 (28%)	1,700 (33%)	539 (19%)	
**Sex**				0.003
Female	2,608 (33%)	1,752 (34%)	856 (31%)	
Male	5,361 (67%)	3,423 (66%)	1,938 (69%)	
**Race**				0.93
Asian	23 (0.3%)	13 (0.3%)	10 (0.4%)	
Black or African American	41 (0.5%)	27 (0.5%)	14 (0.5%)	
Hispanic or Latino	3 (<0.1%)	2 (<0.1%)	1 (<0.1%)	
White	6,692 (84%)	4,358 (84%)	2,334 (84%)	
Other Race	551 (6.9%)	354 (6.8%)	197 (7.1%)	
Missing	659 (8.3%)	421 (8.1%)	238 (8.5%)	
**Practice Type**				0.138
Academic	2,163 (27%)	1,369 (26%)	794 (28%)	
Community	5,688 (71%)	3,732 (72%)	1,956 (70%)	
Both	118 (1.5%)	74 (1.4%)	44 (1.6%)	
**Diagnosed During or After 2017**				<0.001
No	3,703 (46%)	2,258 (44%)	1,445 (52%)	
Yes	4,266 (54%)	2,917 (56%)	1,349 (48%)	
**Insurance at Advanced Diagnosis**				<0.001
Commercial Health Plan	1,398 (18%)	806 (16%)	592 (21%)	
Medicaid	137 (1.7%)	70 (1.4%)	67 (2.4%)	
Medicare (any program)	1,593 (20%)	1,118 (22%)	475 (17%)	
Other government-sponsored/Patient assistance/Self-pay	162 (2.0%)	112 (2.2%)	50 (1.8%)	
Multiple documented	2,834 (36%)	1,914 (37%)	920 (33%)	
Multiple+Other Payer - Type Unknown	1,003 (13%)	655 (13%)	348 (12%)	
Other Payer - Type Unknown	200 (2.5%)	116 (2.2%)	84 (3.0%)	
Unknown	642 (8.1%)	384 (7.4%)	258 (9.2%)	
**Block Group SES index (2015-2019)**				0.033
1 - Lowest SES	815 (10%)	525 (10%)	290 (10%)	
2	1,277 (16%)	794 (15%)	483 (17%)	
3	1,593 (20%)	1,062 (21%)	531 (19%)	
4	1,756 (22%)	1,156 (22%)	600 (21%)	
5 - Highest SES	1,741 (22%)	1,153 (22%)	588 (21%)	
Unknown	787 (9.9%)	485 (9.4%)	302 (11%)	
**ECOG Performance Score**				<0.001
0	3,138 (39%)	2,159 (42%)	979 (35%)	
1	2,291 (29%)	1,412 (27%)	879 (31%)	
2+	908 (11%)	558 (11%)	350 (13%)	
Unknown	1,632 (20%)	1,046 (20%)	586 (21%)	
**Anatomic Site of Melanoma**				<0.001
Head and Neck	1,570 (20%)	1,086 (21%)	484 (17%)	
Lower Limb	961 (12%)	657 (13%)	304 (11%)	
Upper Limb	736 (9.2%)	575 (11%)	161 (5.8%)	
Overlapping	352 (4.4%)	191 (3.7%)	161 (5.8%)	
Truncal	1,774 (22%)	1,122 (22%)	652 (23%)	
Overlapping/Head and Neck	90 (1.1%)	55 (1.1%)	35 (1.3%)	
Truncal/Head and Neck	117 (1.5%)	80 (1.5%)	37 (1.3%)	
Truncal/Lower Limb	129 (1.6%)	87 (1.7%)	42 (1.5%)	
Other	553 (6.9%)	345 (6.7%)	208 (7.4%)	

Unspecified	1,663 (21%)	963 (19%)	700 (25%)	
Unknown	24 (0.3%)	14 (0.3%)	10 (0.4%)	
**Group stage at Initial Diagnosis**				<0.001
0	33 (0.4%)	19 (0.4%)	14 (0.5%)	
I	681 (8.5%)	428 (8.3%)	253 (9.1%)	
II	1,317 (17%)	889 (17%)	428 (15%)	
III	2,074 (26%)	1,536 (30%)	538 (19%)	
IV	2,379 (30%)	1,416 (27%)	963 (34%)	
Not documented	1,485 (19%)	887 (17%)	598 (21%)	
**Had Metastases Outside of the Brain prior to 1L Start**				<0.001
Yes	6,358 (80%)	3,795 (73%)	2,563 (92%)	
No	1,611 (20%)	1,380 (27%)	231 (8%)	
**History of Positive BRAF Status**				<0.001
Yes	2,381 (30%)	1,344 (26%)	1,037 (37%)	
No	2,793 (35%)	1,891 (37%)	902 (32%)	
Unknown	2,795 (35%)	1,940 (37%)	855 (31%)	
